# Antisepsis in Midwifery*Read before the Buffalo Medical Club, March, 1879.

**Published:** 1879-09

**Authors:** Thomas Lothrop


THE BUFFALO

MEDICAL AND SURGICAL JOURNAL.

VOL. XIX.—SEPTEMBER, 1879.—No. 2.

### Original Communications.

### ANTISEPSIS IN MIDWIFERY.*

BY THOMAS LOTHROP, M. D.

THE importance of prophilaxis in the puerperal state, prior as well as subsequent to parturition, is appreciated in proportion as we carefully consider and study the magnitude of the interests involved, and the extent of the responsibility assumed in its management. There can scarcely be a subject of more vital importance than that which investigates the dangers attending the child-bearing woman, and the means to be employed as a protection against such dangers. The wonderful impetus given to antisepsis in surgery by Lister, marks a new epoch in that department; and the diminution in the rate of mortality, after capital as well as minor operations, attest the triumph of the principles he enunciated, and the methods of procedure suggested by his fertile genius. The aim of all antiseptic precautions in surgery, however, is to prevent the microscopic germs or bacteria from contact with the solution of continuity in animal tissue, produced by the knife. The germ theory is the basis of every device and plan, adopted in the methods of this

* Read before the Buffalo Medical Club, March, 1879.

remarkable man, who has left his impress indelibly stamped upon the age in which he lives and the profession he adorns.

In midwifery, another factor, quite as important as that comprehended in the germ theory, is presented in the solution of the problem of morbid phenomena, observed in the puerperal state.

In all ages of the past, the profession have recognized the fact, that the absorption into the living organism of the organic elements of animal tissue, in the process of putrefaction, produces hemorrhagic infiltration, degeneration of parenchymatous organs, softening and mortification, in a word that condition of the system termed typhoid and zymotic. The influence of septic infection in the causation of puerperal disease is acknowledged by every writer in the department of Gynecology and Obstetrics.

Florence Nightingale, in her philanthropic enthusiasm, lays down the following propositions: 1st—"That lying-in patients should be perfectly well as in health. 2d—That no woman ought to die in her lying-in. 3d—That a case of peritonitis or puerperal fever ought never to arise after delivery." Now, while such broad assertions may have been inspired, through self-sacrificing efforts and labors for the amelioration of the sufferings of the race, by the nobleness of that noble woman's nature, the position she assumes involves the question of the origin of disease, which is entirely beyond the scope of human knowledge. The fact must be accepted that woman dies, directly or indirectly as a consequence of the puerperal condition, just as philosophically as we accept the existence of sin, the legacy bequeathed by the transgression of our first parents.

The rate of mortality from the puerperal condition is justly a source of alarm and anxiety to the woman, as the hour of maternity approaches and of opprobrium to the profession, to whose skill the management of this critical period is entrusted. In the department of surgery, to which reference is made above, the diminished rate of mortality is acknowledged to be one of the results of modern scientific research, and of the application of measures, devised by the most assiduous labor, to secure the important end, always held in view by the profession, the preservation of human life. Is there not as wide a field for study in obstetrical science, with the same noble end to be attained, the diminution of the death-rate, and yet more, the prevention of the sundering of sacred ties which the family circle with its increased responsibilities involves? The lying-in chamber calls for the application of like principles and measures to thwart perils, and to counteract dangers, more important than the perils and dangers which accompany the knife of the surgeon in its noble work for humanity.

In 1875 the International Congress of physicians, at Brussels, after a full discussion, adopted the following propositions:

1st. A thorough reform in the help of the lying-in room.

2d. Large lying-in hospitals should be abolished, and their place supplanted by small institutions with separate rooms.

3d. Confinements in the houses of the patients should be encouraged.

4th. These measures will secure a diminished rate of mortality.

This action of the most eminent obstetricians and physicians of the old world is entitled to great respect by the profession on both continents, and should exert a salutary influence in this important department of professional labor. The above propositions were doubtless called forth by the custom, prevailing largely in the great centres of population in Europe, of women of the lower and poorer classes seeking the hospital as a refuge in their approaching confinement. This practice prevails but to a limited extent even in the large cities of this country. The experience of the profession on the continent will lead to a more general effort to bring the rate of mortality in the puerperal state down to the minimum, while the methods of application of protective and sanitary principles will necessarily vary on account of the varying circumstances of locality, habits of people, etc., etc. On the continent the hospitals require a reformation in their principles of construction and methods of arrangement; here the homes, often of the affluent, and more frequently of the humble, must witness whatever of prophilactic and sanitary principles the profession may desire to adopt.

Vital statistics, on this side of the Atlantic, are too imperfectly kept to form a reliable basis, upon which to make even an approximate estimate of the rate of mortality from the puerperal state in this country. Reference must be made to the careful study of this question in Europe, where the subject in all its phases has received special attention.

England is said to furnish the most reliable data, the laws regulating its vital statistics being better enforced than on the continent. In 1847, it is stated in the Registrar General's Report that the mortality in child-birth in England and Wales, was 1 in 167, and in 1856 the death-rate had fallen to 1 in 227 cases.

Mathews Duncan, in his work on the "Mortality of Child-bed and Maternity Hospitals," found the total mortality during the first six weeks after confinement in *home practice* to be 1 in 107 in Edinburgh and Glasgow in 1855.

McClintock * secured valuable data, by taking the house practice of nine entirely reliable physicians who had a total of 16,774 cases, with 45 deaths from accidents in labor, 52 from puerperal fever, and 34 from non-puerperal diseases, or one death in every 128 cases. The same writer publishes the results in his private practice of 652 cases, with 6 deaths, or 1 death in 108 cases; and with great assiduity he has gathered from other prominent obstetricians 16,108 cases, with 120 deaths, or 1 in 134. These cases were among the middle and upper ranks of society.

Duncan furnishes the result in his own private practice as 8 deaths in 736 cases, or 1 in 92.

---

* Dublin Quarterly Journal, 1869.

After a very careful examination of the reports, returned by the Registrar General of different countries, Duncan furnishes the following valuable facts :

| | | |
|---|---|---|
| Paris, in 1860, one death in | - | 120 cases. |
| St. Petersburgh, " | - - | 112 " |
| Dublin, " | - - | 86 " |
| Edinburgh, (1860 to 1865) one death in | | 122 " |

In Copenhagen, outside of the Maternity Hospital, 108,737 women were delivered during the 25 years from 1850 to 1874, 885 of whom died from puerperal fever alone, or 1 in 123.

Since 1873 we are furnished with direct means of ascertaining the death-rate, not only from puerperal fever, but also from other causes. Since that date all deaths, due to parturition, and the puerperal state, are registered in two classes, one for puerperal fever, and the second for child-birth and child-bed, exclusive of puerperal fever.* From these statistics we find 127 deaths from puerperal fever and 54 from other causes. This makes the death-rate in Copenhagen in the hospital and in the city at large to be 1 in 86 cases.

Summarizing the data from these various sources, excluding those that cannot be relied on, the average mortality is found to be 1 in a little over 100 cases.

| | | |
|---|---|---|
| Edinburgh and Glasgow in 1855, | - | 0.93 per ct. |
| Paris, St. Petersburgh, Dublin and Edinburgh, | - - - | 0.92 " |
| Copenhagen, | - - - - | 1.16 " |
| Duncan, McClintock, and other prominent Obstetricians, | - - | 0.75 " |
| Average, | - - - - - | 0.94 per ct. |

In view of the above facts, an eminent philanthropist makes the following pertinent remark :

" One feels disposed to ask whether it can be true that in the hands of educated accoucheurs, the inevitable fate of women,

* Physicians' Weekly Journal.

undergoing not a diseased, but an entirely natural condition at home, is that one out of every 100 must die."

The rate of mortality in many of the hospitals of the old world may be of interest in this connection. In the Lariboisiere, in Paris, there has been an average mortality of nearly 8 per cent. of lying-in women. In one service of the lying-in hospital in Vienna the average death-rate for the six years, from 1841 to 1846, was almost 10 per cent. During the four years from 1860 to 1864 in the Maternité of Paris, the average mortality was 11 per cent. The Rotunda of Dublin, which is more than a century old, and has had over 190,000 deliveries, has experienced a death-rate of 1 in 72 cases, and for long periods less than 1 in 100 cases. In this hospital, for the seven years, from 1869 to 1875, the death-rate was 1 in 48 cases, or 2.1 per cent. In the Royal Lying-in Hospital of Dresden, considered the finest on the continent, the mortality was 1.1 per cent. in 1869 and 1870; 0.9 per cent. in 1871, and 5.2 per cent. in 1872, the latter being the result of an epidemic. Under the service of Winckel, who became physician-in-chief in 1872, the death-rate was reduced again to 2.3 per cent. in 1873; 1.2 per cent. in 1874, and 1.3 per cent. in 1875. These results were obtained only by the adoption of the most stringent sanitary measures.

The New York lying-in institutions furnish valuable data upon this subject. In the Lying-in Asylum, 85 Marion street, the death-rate from 1856 to 1866 was 1.1 per cent. In the New York Infant Asylum, 24 Clinton Place, for the five years from 1872 to 1876, there were 418 cases and only 5 deaths, or 1.2 per cent. The Infirmary for Women and Children, No. 5 Livingston Place, during the nineteen years, from 1858 to 1876, 1350 women were confined, with a death-rate of 1.2 per cent.

The Nursery and Child's Hospital, corner Lexington Avenue and Fifty-First street, from 1865 to 1876, had 1479 confinements and 60 deaths, or 4.1 per cent.

In the Charity Hospital, on Blackwell's Island, for the two and a half years, from 1874 to 1876, there were 1381 births, and 36 deaths, or 2.6 per cent.

The State's Emigrant Hospital on Ward's Island, for nine years, from 1868 to 1876, there were 3766 confinements, and 99 deaths from all causes, or 2.9 per cent.

In referring to the statistics of diseases to which lying-in women succumb, it will be observed that puerperal fever is the most prevalent and fatal. For instance, in Copenhagen, in the years 1873, '74, '75, there were 127 deaths from puerperal fever, to 54 from all other causes. This proportion will vary at different times, but the fever will always be in excess of other diseases. This is important in considering the question of prophilaxis. The pathological conditions, common to this period, and the necessity for the adoption of preventive measures to counteract the danger of septic infection, are apparent. Puerperal septicaemia and pyaemia may also be classed in this category. Diseases arising from traumatism, as a primary condition, have also an important relation with this question.

It is admitted, also, that puerperal fever may be auto-genetic in its origin. Poisonous materials may be generated in the system under the strain of labor; the blood may be surcharged with noxious matter, resulting from tissue changes, the materies morbi being unknown, and producing a disease as distinct as typhus or typhoid fever.

Puerperal fever may also arise from septic infection. Under the combined influences of traumatism, and of the blood impaired by the tissue changes of pregnancy and parturition, and also of the decomposing debris of placenta and blood-clots in the uterus, septic materials are thus supplied in the uterine cavity itself, and through the absorbent surface offered by the mucous membrane, are taken up and transmitted to the system. This factor, allied to the conditions producing puerperal septicaemia, is of special importance in view of prophilaxis. These hetero-genetic causes imply a peculiar susceptibility in the system, which must be met by other than local measures, although local measures are not without a marked influence. While there are conditions of the system local and general, predisposing to the complications often met with in the puerperal state, the great exciting cause, paramount to all others in the causation of these peculiar pathological phenomena, is parturition acting upon the systemic changes, which precede the completion of utero-gestation.

The structure and functions of the mucous membrane lining the uterine cavity, and, to a limited extent, the vagina, possess peculiar significance in considering the diseases, resulting from the puerperal condition, subsequent to the completion of the parturient effort. That its structure undergoes changes in its development to fit it for the special office the uterus holds in the economy of nature, has been fully demonstrated by Engelman and others. Not the least important element in its structure are the small tubular glands, generally running perpendicular to the surface, often bifurcating in their lower third, and then becoming imbedded in the substratum of connective tissue. According to Engelman there is an entire absence of submucous areolar tissue. Now under the stimulus of menstruation, and to a yet greater degree, of pregnancy, these glands are many times enlarged, the increase in their length being greater than the increase in the thickness of the mucous membrane in which they are imbedded, while there is an intense vascular injection of even the finest vessels. The orifices of these glands become more widely separated from each other as they are developed, and are like funnel-shaped depressions in the thickened mucous membrane.

The provision which nature makes to prepare for the impregnated ovum a safe repository in which the successive stages of its development can go on uninterrupted by external circumstances, becomes a source and avenue of danger as soon as the fully developed ovum vacates its retreat, and begins an independent existence.

It would be foreign to the purpose of this paper to consider the special office of the glandular structures in utero-gestation, and they are referred to in this connection, to direct attention to their power of absorption, which is one of the functions of the membrane, of which they form an important element. The great development of the glandular structure has its purpose in meeting the demands of osmosis, set up between the maternal and foetal circulation, and its power of absorption is correspondingly increased with the development of its histological elements.

The increased extent of this absorbing surface, due to the increased length and width of the uterus, and the influx of blood to all the pelvic organs under the special stimulus of pregnancy, constitutes an important element of danger. It is well to lay stress upon these facts, and to direct attention to the danger thus entailed upon the parturient woman, which is not sufficiently considered by many physicians in obstetrical practice. Too much dependence is placed upon the excretory function of the mucous membrane lining the uterine cavity, while the function with which it is also invested, of absorbing fluids, the debris of tissue, most of which is septic material, always abundant during the disintegrating process, instituted by nature to return to the status existing prior to impregnation, is lost sight of. The result is the frequent occurrence of septic and zymotic disease, making too often the lying-in room the scene of suffering and death.

Considerations, such as are presented above, bearing upon the mortality of the puerperal state, subsequent to parturition, and the physiological and pathological causes leading thereto, suggest naturally the inquiry: "What measures can be adopted to diminish the dangers and the mortality of the lying-in chamber?" It is to the practical aspect of this question, omitting all theoretical considerations, that the writer directs attention, believing the accoucheur should bestow the same careful attention to minute details in parturition that the surgeon adopts to thwart the contact of the invisible germs floating in the atmosphere and following every movement of his knife.

As a practical illustration of the influence of antiseptics in midwifery, I beg to refer again to the lying-in asylum of Copenhagen,* in which the plan has been fully tried. During 22 years (1822 to 1843), 21,149 women were confined there, of whom 1,096 died of puerperal fever, or 1 in 19. Influenced by the remarkable results obtained in the surgical service by the adoption of Lister's method of operating and of dressing wounds, Stadfeldt, the physician-in-chief, introduced a strict preventive antiseptic treatment. A small room was set apart for deliveries, and from 2 to 6 hours after delivery, the patient is brought in the bed in which she has been delivered into one of the rooms, opening on the other part of the corridor. Inasmuch as the establishment is a school for the training of midwives, the pupil goes with her patient from the delivery apartment, to the lying-in department, and when the patient leaves, the nurse takes a bath, and her body and clothes are disinfected in the following way: connected with the window of a small room is a *hose* of sufficient size to cover the head, allowing space for free respiration, while her person and clothes are subjected for a quarter of an hour to the fumes of sulphurous acid. Before and after digital examination per vagina, the hands are washed with a solution of carbolic acid, and all instruments used in operation are disinfected in this carbolized water. If possible the confinement takes place under a spray of the same disinfectant, vaginal injections with carbolized water (1 pt. to 125) are used in every patient twice a day; all lesions are dressed with carbolized ointment, and the external genitals covered with carbolized oil, which, of late, has been replaced with salicylic acid in ten parts of wheat flour, powdered over the parts two or three times daily. The patient rests on a sack, filled with chaff, both sack and contents being burned when the patient is discharged. Each patient is allowed a pillow of hair, and also blankets, and if the delivery has been attended with anything abnormal, these articles are thoroughly cleansed. The room remains empty after each confinement, and is often disinfected. The same bed-pans, syringes, and catheters, are never used for the sick and the well. The water-closets are disinfected

* Gynecological Transactions, 1877.

every day. The after-births and soiled clothes are immediately thrown into a pail containing a strong solution of chlorinated lime and burned the next morning.

The result of this antiseptic treatment accomplished all that was expected of it. During the 20 years, from 1850 to 1869 inclusive, there were 21,675 deliveries, of whom 815 died of puerperal fever, or 1 in 26, while during the 5 years, from 1870 to 1874 inclusive, in which special attention was directed to antisepsis, there were 5,304 deliveries, with 61 deaths from puerperal fever, or 1 in 87; the highest mortality being 1 in 75, the lowest 1 in 170.

These figures show the relative value of antiseptic measures in this hospital alone, in which many of the conditions existed, which brought about the terrible mortality of 1 in 19 cases, reported prior to 1845.

The value of antisepsis in puerperal septicaemia will be best demonstrated by the report of a case.

Mrs. A., aged 35 years, a native of the United States, was confined May, 1875, after a tedious and protracted labor; the placenta came away soon after, and seemed to be accompanied with all the membranes in which the foetus had been previously invested. The progress of this case was uniformly good until the 10th day after delivery, when abdominal pain and difficult micturition came on. The lochia were scanty and of a fetid odor; temperature 103; pulse 107; morphia ½ grain was injected; warm fermentation applied to hypogastrium, the urine drawn with the catheter, the vagina thoroughly washed with carbolized water, and opiates repeated to maintain rest and freedom from pain. In the evening of the same day there was but little pain, loss of appetite, dry tongue, some tympanitis, temperature 105, pulse 114. The injections of carbolic acid were repeated, the nurse being directed to carry the nozzle of the syringe as high as possible, and throw the antiseptics as near the os uteri as it was possible to do. At near midnight I was summoned in haste, found the patient in a severe chill, with no pain, face flushed, the pupil's natural and but little tenderness over the abdomen; the pulse was 150 per minute, the temperature 107. With a flexible male catheter attached to a Davidson Syringe, I carried it up to the fundus of the uterus and thoroughly washed out the uterine cavity with carbolized water at a temperature of 99°. Brandy was freely given with tr. opii as an adjuvant. After the injection the temperature fell to 104, and the pulse to 123.

At 2:00 A. M., pulse 115, temperature 102.8, and the patient warm and sweating profusely.

At 3:00 A. M. I left her with a pulse of 115, temperature 102.5, and with no pain.

At 10.00 A. M. following day, pulse 105, temperature 102.

8 P. M., temperature 103.1, and pulse 112.

From this date the patient convalesced without unfavorable symptoms, and in due time was discharged.

The marked influence of antiseptic injections into the uterine cavity, in the above patient, may have been almost exceptional, but the case elucidates more fully than words can express the advantages of the application of these principles of treatment in the puerperal state, and it is for this purpose here reported. In looking over the literature of the subject, I have found cases reported of even greater professional interest and attended with equally satisfactory results.

Referring to intra-uterine injections in puerperal septicaemia, Dr. Fordyce Barker, who is one of the clearest thinkers the medical profession of this country has yet produced, says: "I should certainly not hesitate to recommend them, if the history of the case and the symptoms indicate that the septicaemia was the consequence of, or was complicated with, endometritis."

There have been fatal cases attributed to this method of medication, but it may be justly questioned whether the care required in the minute details of the operation was duly observed. The injection into the uterine cavity of air is always hazardous and frequently fatal, and it is not too much to surmise that much of the danger resulting from intra-uterine injections may have arisen from its presence in the syringe, and its careless introduction into the uterine sinuses.

It would be beyond the scope of a single paper to refer to other complications of the puerperal state, often met with in the experience of the obstetrician. The application of antiseptics to puerperal metritis and peritonitis and phlebitis, to pelvic peritonitis and cellulitis, and other diseases complicating this condition must be inferred from what has been said above.

While there is a great diversity of opinion in regard to the pathology of milk fever, many of the most prominent continental pathologists and obstetricians attribute the feverish excitement, occurring on the second or third day after parturition, to proceed from the bruising and injury of the genital canal during the progress of the foetus, and the subsequent absorption of septic matter from the lochia. In other words it is allied to wound fever. Winckel and Kiwisch support this view. Dr. D'Espine, a French writer, takes this position. The theory of wound fever seems to me so well proven, and substantiated by eminent authority that the application of antisepsis to the period of convalescence in the lying-in woman, at which this feverish excitement usually occurs, seems to be peculiarly indicated. In my own experience the effects of antisepsis in preventing the occurrence of the fever, have borne out the theory of the eminent writers to whom reference is made above; and the limited experience usually given to the general practitioner in the cases annually attended, is deserving of consideration by his professional brethren, who seek to secure additional safeguards to those who entrust their accouchement to his skillful management.

The facts presented above, may be summarized as follows:

1st. The puerperal state, prior as well as subsequent to parturition, calls for the rigid application of prophilactic principles and measures.

2d. The success of Lester's methods of antisepsis in surgery, calls for the employment of measures founded on the same principles in midwifery, especially in view of the additional factor (septic infection) encountered in the puerperal state.

3d. The average mortality, 0.94 per cent, or one death in little over 100 cases, the most accurate data furnished by reliable European authority, demands the serious consideration of the medical profession, and calls for earnest efforts to secure better results.

4th. Antisepsis in the Maternity Hospital of Copenhagen, affords a practical demonstration of the utility of such measures in obstetric practice.

5th. Puerperal fever can be limited by the free use of antisepsis.

6th. Septic infection may be controlled in many cases by the application of antiseptics to the intra-uterine mucous membrane.

7th. Milk fever, being essentially a wound fever, due to septic infection through injury done to the vaginal and uterine surfaces during parturition, is prevented by antisepsis.

Lastly. The considerations thus imperfectly presented, direct special attention to two important propositions. 1st, The large death-rate. 2d, Measures of prevention.

With these two points in view, the writer presents incontrovertible facts, such as have been within reach, avoiding useless theorizing, while asking for them the candid consideration the vital importance of the subject demands.

Should these feeble suggestions lead to the employment of the means above indicated, and the saving of even one, however humble and lowly, who has experienced "The strange and passionate precipitance of maiden into motherhood," the fullest purpose of the writer will be attained.

